# Let’s get started with targeted temperature management

**DOI:** 10.1186/1471-227X-15-S1-A1

**Published:** 2015-06-24

**Authors:** Fabio Silvio Taccone

**Affiliations:** 1 Department of Intensive Care, Hopital Erasme, Université Libre de Bruxelles, Brussels, Belgium

## 

Cardiac arrest (CA) remains a major cause of death and severe disability worldwide. The ischemic process that follows the cessation of cerebral perfusion and oxygenation, which is further worsened by the reperfusion injury occurring after the return of spontaneous circulation, can lead to severe hypoxic brain damage, resulting in a high rate of poor neurological recovery among CA survivors.

The use of mild therapeutic hypothermia, or targeted temperature management (TTM) as recently suggested [1], has been recommended in CA patients since the publication of two randomized clinical trials in 2002, the results of which demonstrated a significant improvement in neurologically intact survival for comatose CA patients presenting with ventricular fibrillation or ventricular tachycardia [1,2]. Current guidelines suggest that mild therapeutic hypothermia should also be considered in patients presenting with other rhythms, although this has been less well studied [3].

In experimental studies, TTM provided significant cardiac and neurological protective effects through different pathways. Hypothermic mechanisms providing myocardial protection include, amongst all, improved energy production during ischemia, increased calcium sensitivity of myocytes, regulation of mitochondrial oxidative phosphorylation and preserved myocardial vascular autoregulation [4,5]. All of these protective mechanisms would result in increased myocardial contractility. After a post-anoxic injury, TTM may also protect cerebral function through reduced release of excitatory (that is, glutamate and dopamine) neurotransmitters, attenuation of reactive oxygen species production, preservation of the blood–brain barrier, protection of cerebral microcirculation and decrease in intracranial pressure [6,7]. As several pathways are involved in the pathogenesis of extended post-anoxic brain damage, TTM can be considered as a general and nonspecific neuroprotective strategy, which may efficiently attenuate and mitigate most of these mechanisms and potentially improve patients’ neurological outcome. Interestingly, recent studies have underlined not only that the hypothermic phase is important in this process, but that strict control of the patient’s temperature during the first 3 days since hospital admission (that is, rapid achievement of target temperature, a precise control of temperature during the maintenance phase, a slow and controlled rewarming and avoidance of fever for 48 to 72 hours) are key components to enhance TTM effectiveness after post-anoxic brain injury [8].

## Financial disclosure

FST received a research grant from Benechill in 2010 for animal research on IAHT.

## Declaration

This abstract and supplement was proposed, developed and commissioned by BMC Emergency Medicine and was funded by an educational grant from C. R. BARD, NJ, USA. The published abstract was independently prepared by the author. C. R. BARD had no input into the content.
